# Spontaneous Rupture of Splenic Artery Aneurysm during the First Trimester of Pregnancy: Report of an Extremely Rare Case and Review of the Literature

**DOI:** 10.1155/2012/528051

**Published:** 2012-09-17

**Authors:** Theodoros Pavlis, Charalampos Seretis, Stavros Gourgiotis, Paraskevi Aravosita, Christina Mystakelli, Stavros Aloizos

**Affiliations:** Intensive Care Unit, “Mitera” Obstetric & Gynecological Hospital Athens, 6 Erithrou Stavrou Street, Amarousion, 151 23 Athens, Greece

## Abstract

Splenic artery aneurysm (SAA) occurs predominantly in women and the majority of them are asymptomatic until rupture. In cases of spontaneous rupture of an SAA, maternal and fetal mortality rates remain extremely high. Furthermore, the spontaneous ruptures of SAAs predominantly appear during the third trimester of pregnancy. We present the third known case of spontaneous SAA rupture during the first trimester of pregnancy, which manifested as sudden hypovolemic collapse and was successfully confronted with combined aggressive resuscitation and emergency surgical operation.

## 1. Introduction

Spontaneous rupture of splenic artery aneurysm (SAA) is a catastrophic and potentially life-threatening complication of pregnancy, for both the pregnant mother and fetus; maternal and fetal mortality rates remain extremely high, up to 70% and 90% respectively [1], underlining the necessity of immediate intervention.

The mechanisms which are involved in the formation of this vascular defect still remain unclear. Various theories have been proposed for the explanation of this phenomenon, mainly considering hemodynamic and hormonal alterations in the late stages of pregnancy [2]. Although the risk of rupture mainly exists in the third trimester of pregnancy [2], up to this date, only two cases of SAA rupture during the first trimester of pregnancy have been reported in the literature [3, 4]. 

We present the third known case of spontaneous SAA rupture during the first trimester of pregnancy, which manifestated as sudden hypovolemic collapse and was successfully confronted with combined aggressive resuscitation and emergency surgical operation. Diagnostic evaluation and management are discussed, along with a review of the literature.

## 2. Case Presentation

A 34-year-old multiparous pregnant women was referred to the emergency department, complaining of diffuse abdominal pain and episodes of vomiting, which had started approximately three hours prior to her admission. She was 8 weeks pregnant, with an unremarkable past medical history.

The patient was hemodynamically unstable; her blood pressure was 90/60 mmHg, pulse rate 145 beats/minute, SpO_2_ 95%, and body temperature 38.1°C. The hemogram revealed a white cell count of 11,700/mm^3^ with 91% neutrophils, a hemoglobin level of 4.2 g/dL, and a platelet count of 98.000/mm^3^. All biochemical laboratory tests were unremarkable. The arterial blood gas examination demonstrated the establishment of severe acidosis, with a pH value at 6.9. Physical examination revealed a distended abdomen with evident peritoneal irritation, guarding, and rebound tenderness. There was absence of bowel sounds and vaginal bleeding. The patient was in distress. Emergency abdominal ultrasound (U/S) revealed the presence of a massive hemoperitoneum.

Despite the aggressive resuscitation efforts with intravenous crystalloid fluids and norepinephrine, the patient's mental status was gradually deteriorating and the abdominal pain was becoming more intense. The patient soon lost her consciousness. She was immediately intubated and taken to the operating room, where an exploratory laparotomy was performed. We did not perform whole abdominal enhanced computed tomography (CT) before the laparotomy due to the fact that the patient was unstable. Our main concern was to save the patient which was the reason the emergency exploratory laparotomy was performed.

A surgeon was called and he operated on the patient. The exploration of the peritoneal cavity failed to reveal a gynecological origin of the massive hemoperitoneum, excluding as a possible cause of the bleeding the presence of an ectopic pregnancy, which had been at the moment the speculated cause of hemorrhage. Surprisingly, careful examination indicated that the lesser sac was full of blood. The gastrocolic ligament and the transverse mesocolon were incised to approach an SAA which was found with a 5 mm-lengthed rupture, located at the distal third of the artery's length. Proximal ligation of the splenic artery and splenectomy were performed, resulting in adequate control of the bleeding source. The patient was transferred to the intensive care unit for further support. In total, she received 8 units of packed red cells, 4 units of platelets, and 8 units of FFP. She made an uneventful recovery and was discharged on the 10th postoperative day. She was commenced on prophylactic penicillin V and received polyvalent pneumococcal, meningococcus, and Hemophilus influenza vaccines. Unfortunately, the patient did not continue with the pregnancy.

## 3. Discussion

The vast majority of SAAs, being smaller than 2 cm, remains asymptomatic and is generally encountered as autopsy findings [5]. Nevertheless, up to date, more than 400 cases of ruptured SAAs have been reported in the international literature, with approximately 30% of these during pregnancy [6]. Moreover, almost all cases of ruptured SAAs in pregnancy have occurred during the third trimester [6]. Thus, it is evident that the physical history of pregnancy gradually increases the risk of rupture of SAA. Apart from the late stage of pregnancy, other risk factors include multiparity, portal hypertension, atherosclerosis, and medial fibrodysplasia [7].

One of the mechanisms promoting the vascular defect in splenic arteries during the late stages of pregnancy seems to be the escalating increase of the circulating estrogens and progesterone during pregnancy [8]. The elevation of the levels of these hormones has been associated with promotion of various structural alterations in the arteries, such as the disruption of the internal elastic lamina, fragmentation of elastic fibers, degeneration of smooth muscle fibers, and failure of elastin formation [9]. Additionally, it appears that the elevated levels of relaxin throughout the third trimester of pregnancy may affect the elasticity of the splenic artery wall [10]. Especially in case of multiparity, the repeated exposure to these hormonal shifting could explain the increased incidence of rupture of SAA in this group of pregnant women. It is also assumed that hemodynamic changes which occur during the late stages of pregnancy are implicated in the etiology of SAA ruptures during this period. More specifically, due to the fact that the increased size of the uterus tends to compress the aorta and the iliac arteries, resulting in higher flow in the splenic artery, the development of the SAA is enhanced; moreover, the increases of blood volume and cardiac output, along with the relative portal congestion, can definitely contribute to the formation of SAAs [2].

In terms of clinical manifestations, the rupture of SAA is undoubtedly presented as an acute abdomen, with localization of pain more frequently in the epigastrium or the left hypochondrium, accompanied by vomiting and derangement of the vital signs, compatible with developing hemodynamic shock. The physical course of the rupture of SAA can either consist of one stage, leading to dramatic collapse as a result of inability of self-containment of bleeding, or it can present in a two-stage sequence, when hemorrhage would be initially tamponated in the lesser sack by clots blocking the foramen of Winslow [10]. Even in the latter case, when the physician may have an advantage in terms of time availability, the gradual increase of pressure in the lesser sac would be suddenly followed by a rupture into the greater sac and lead to massive intraperitoneal bleeding.

The key of effective management of a ruptured SAA is increased clinical suspicion, combined with accurate implementation of the diagnostic means available, particularly abdominal U/S and angiography, if permitted by the hemodynamic condition of the patient. Concerning intervention options, apart from the need of initial aggressive resuscitation, embolization of the aneurysm or emergency surgical operation with ligation of the ruptured aneurysm, followed or not by splenectomy, stand for the most realistic treatment options [2, 10].

We presented a case of successful surgical management of a ruptured SAA, which occurred during the first trimester of pregnancy. Searching the English literature, we encountered only two similar cases [3, 4], in which the initial diagnosis was suspected rupture of an ectopic pregnancy, similar to our case, and the patients underwent exploratory laparotomy in order to control the source of bleeding. In these two cases, proximal ligation of the splenic artery alone with preservation of the spleen was performed, in contrast to our reported case. Unfortunately, these two patients died due to cardiac arrest during the early postoperative period. Nevertheless, it is evident that the dominating element in all cases was the unexpected final cause of the hypovolemic shock, as rupture of SAA was not considered in the initial differential diagnosis, presumably due to its firm association with more advanced stages of pregnancy.

In conclusion, a hemoperitoneum in early pregnancy should not a priori be considered as a result of ectopic pregnancy alone. It is therefore important to increase awareness of the possibility of a spontaneous rupture of an SAA in any pregnant woman who presents with severe upper abdominal pain and hemodynamic instability.

## References

[1] Hillemanns P, Knitza R, Muller-Hocker J (1996). Rupture of splenic artery aneurysm in a pregnant patient with portal hypertension. *American Journal of Obstetrics and Gynecology*.

[2] Parangi S, Levine D, Henry A, Isakovich N, Pories S (2007). Surgical gastrointestinal disorders during pregnancy. *American Journal of Surgery*.

[3] Groussolles M, Merveille M, Alacoque X, Vayssiere C, Reme JM, Parant O (2011). Rupture of a splenic artery aneurysm in the first trimester of pregnancy. *Journal of Emergency Medicine*.

[4] Chookun J, Bounes V, Ducassé JL, Fourcade O (2009). Rupture of splenic artery aneurysm during early pregnancy: a rare and catastrophic event. *American Journal of Emergency Medicine*.

[5] Lang W, Strobel D, Beinder E, Raab M (2002). Surgery of a splenic artery aneurysm during pregnancy. *European Journal of Obstetrics Gynecology and Reproductive Biology*.

[6] Abbas MA, Stone WM, Fowl RJ (2002). Splenic artery aneurysms: two decades experience at Mayo Clinic. *Annals of Vascular Surgery*.

[7] Messina LM, Shanley CJ (1997). Visceral artery aneurysms. *Surgical Clinics of North America*.

[8] Dave SP, Reis ED, Hossain A, Taub PJ, Kerstein MD, Hollier LH (2000). Splenic artery aneurysm in the 1990s. *Annals of Vascular Surgery*.

[9] Sadat U, Dar O, Walsh S, Varty K (2008). Splenic artery aneurysms in pregnancy-a systematic review. *International Journal of Surgery*.

[10] Kunz MN, Pantalone D, Borri A (2003). Management of true splenic artery aneurysms. Two case reports and review of the literature. *Minerva Chirurgica*.

